# Evaluation of integrated care services in Catalonia: population-based and service-based real-life deployment protocols

**DOI:** 10.1186/s12913-019-4174-2

**Published:** 2019-06-11

**Authors:** Erik Baltaxe, Isaac Cano, Carmen Herranz, Anael Barberan-Garcia, Carme Hernandez, Albert Alonso, María José Arguis, Cristina Bescos, Felip Burgos, Montserrat Cleries, Joan Carles Contel, Jordi de Batlle, Kamrul Islam, Rachelle Kaye, Maarten Lahr, Graciela Martinez-Palli, Felip Miralles, Montserrat Moharra, David Monterde, Jordi Piera, José Ríos, Nuria Rodriguez, Reut Ron, Maureen Rutten-van Mölken, Tomas Salas, Sebastià Santaeugenia, Helen Schonenberg, Oscar Solans, Gerard Torres, Eloisa Vargiu, Emili Vela, Josep Roca

**Affiliations:** 1 Hospital Clinic de Barcelona, Institut d’Investigacions Biomèdiques August Pi i Sunyer (IDIBAPS), Universitat de Barcelona, Barcelona, Spain; 2 Center for Biomedical Network Research in Respiratory Diseases (CIBERES), Madrid, Spain; 3 CAPSBE. Consorci d’Atenció Primaria de Salut. Barcelona Esquerra, Barcelona, Spain; 4 Royal Philips Netherlands BV acting through Philips Homecare, Boeblingen, Germany; 5 Area d’Atenció Sanitària, Servei Català de la Salut, Barcelona, Catalonia Spain; 6 Chronic Care Program. Ministry of Health, Generalitat de Catalunya, Barcelona, Catalonia Spain; 7 Respiratory Department, Institut de Recerca Biomedica (IRBLeida), Lleida, Spain; 8 Department of Economics, University of Bergen, Bergen, Norway; 9 Assuta Medical Centers, Tel Aviv-Yafo, Israel; 10 Department of Epidemiology, University of Groningen, University Medical Center Groningen, Groningen, the Netherlands; 11 Eurecat. Technological Center of Catalonia, Barcelona, Catalunya Spain; 12 Agència de Qualitat i Avaluació Sanitàries de Catalunya (AQuAS), Barcelona, Catalonia Spain; 13 Institut Català de la Salut, Serveis Centrals, Barcelona, Catalonia Spain; 14 Badalona Serveis Assistencials (BSA), Badalona, Catalonia Spain; 15 Medical Statistics Core Facility, Institut d’Investigacions Biomèdiques August Pi i Sunyer (IDIBAPS) and Hospital Clinic, Barcelona, Spain; 16 Biostatistics Unit, Faculty of Medicine, Universitat Autònoma de Barcelona, Barcelona, Spain; 17 School of Health Policy and Management, Erasmus University Rotterdam, Rotterdam, the Netherlands; 18 Institute for Medical Technology Assessment, Erasmus University Rotterdam, Rotterdam, the Netherlands; 19 Central Catalonia Chronicity Research Group (C3RG), University of Vic – Central University of Catalonia, 08500 Vic, Spain

**Keywords:** Chronic patients, Integrated care services, Multimorbidity, Service transferability, Home hospitalization, Prehabilitation, Digital tools, Implementation science, Risk assessment, Multi-criteria decision analysis

## Abstract

**Background:**

Comprehensive assessment of integrated care deployment constitutes a major challenge to ensure quality, sustainability and transferability of both healthcare policies and services in the transition toward a coordinated service delivery scenario. To this end, the manuscript articulates four different protocols aiming at assessing large-scale implementation of integrated care, which are being developed within the umbrella of the regional project Nextcare (2016–2019), undertaken to foster innovation in technologically-supported services for chronic multimorbid patients in Catalonia (ES) (7.5 M inhabitants).

Whereas one of the assessment protocols is designed to evaluate population-based deployment of care coordination at regional level during the period 2011–2017, the other three are service-based protocols addressing: i) Home hospitalization; ii) Prehabilitation for major surgery; and, iii) Community-based interventions for frail elderly chronic patients. All three services have demonstrated efficacy and potential for health value generation. They reflect different implementation maturity levels. While full coverage of the entire urban health district of *Barcelona-Esquerra* (520 k inhabitants) is the main aim of home hospitalization, demonstration of sustainability at Hospital Clinic of Barcelona constitutes the core goal of the prehabilitation service. Likewise, full coverage of integrated care services addressed to frail chronic patients is aimed at the city of Badalona (216 k inhabitants).

**Methods:**

The population-based analysis, as well as the three service-based protocols, follow observational and experimental study designs using a non-randomized intervention group *(integrated care)* compared with a control group *(usual care)* with a propensity score matching method. Evaluation of cost-effectiveness of the interventions using a Quadruple aim approach is a central outcome in all protocols. Moreover, multi-criteria decision analysis is explored as an innovative method for health delivery assessment. The following additional dimensions will also be addressed: i) Determinants of sustainability and scalability of the services; ii) Assessment of the technological support; iii) Enhanced health risk assessment; and, iv) Factors modulating service transferability.

**Discussion:**

The current study offers a unique opportunity to undertake a comprehensive assessment of integrated care fostering deployment of services at regional level. The study outcomes will contribute refining service workflows, improving health risk assessment and generating recommendations for service selection.

**Trials registration:**

NCT03130283 (date released 04/06/2018), NCT03768050 (date released 12/05/2018), NCT03767387 (date released 12/05/2018).

**Electronic supplementary material:**

The online version of this article (10.1186/s12913-019-4174-2) contains supplementary material, which is available to authorized users.

## Background

Core elements of integrated care (IC) are connectivity, alignment and collaboration within and between the cure and care sectors. The goal is to enhance quality of care and quality of life, consumer satisfaction and system efficiency for patients suffering from chronic disorders, that need multiple services, providers and settings in different levels of care [1–3]. Useful approaches [4] have identified two main systemic levels (i.e. horizontal and vertical) at which integration of health and social care sectors can occur. Horizontal integration links community-based services while vertical integration brings together specialized and primary care under one functional (or structural) management umbrella through shared care agreements framed into well-defined service workflows.

Since early 2000’s, large scale implementation of IC is being strongly promoted by relevant international agencies and governments [5, 6] because of its high potential to effectively address the healthcare and societal challenges generated by population ageing and unhealthy lifestyles. However, several aspects implicit in the transition towards real care coordination scenarios, must be taken into account and properly solved to ensure adoption. First, since IC services are applied to complex patients and in evolving settings, the need for flexible standardization of the interventions, as well as changes in the roles of patients and health professionals is a must. Second, the coordination between several stakeholders and/or healthcare tiers often requires profound organizational adaptations which, in turn, involve the need for novel business models and reimbursement incentives to drive management change. Last but not least, quickly evolving digital technologies are facilitating coordination and personalization of care, as well as complex data management, but extensive adoption of digital health supporting IC needs to be accelerated.

All of the above factors contribute to explain the difficulties encountered in the process of standardization of IC assessment. Over the past several years, evaluation of well-established IC programs, alongside pilot experiences, has been undertaken in several countries with mixed results [7–9]. Overall, these experiences have contributed to the generation of a series of general recommendations on evaluation of IC with focus on service transferability across geographical sites aimed at fostering regional scalability [4, 10]. It is of note, however, that application of these recommendations for a comprehensive assessment of deployment of IC services in real-life scenarios is clearly an unmet need.

**Fig. 1:**
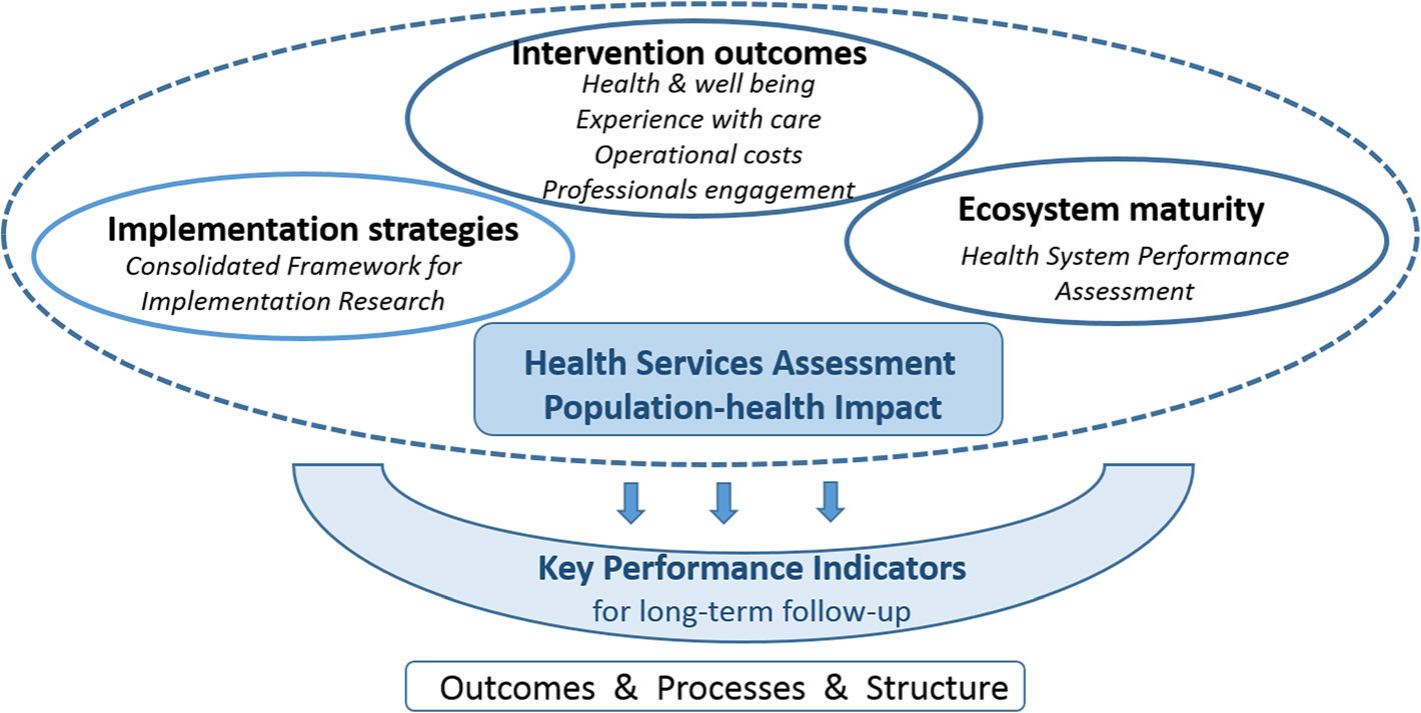
The figure depicts the main elements of the structured evaluation framework that articulates the four assessment protocols described in the current report. The proposed comprehensive assessment of integrated care services includes their impact at population level. A core component of the assessment protocols includes the identification of Key Performance Indicators (KPI) useful for long-term follow-up of health services after adoption encompassing three dimensions: health outcomes, processes and structure

The current manuscript aims to describe a structured evaluation framework (Fig. 1) that articulates four comprehensive assessment protocols covering both vertical and horizontal levels of integration. One assessment protocol reports a population-based assessment of outcomes from past and current Catalan Health Plans, 2011–2015 [11] and 2016–2020 [12], respectively, whereas the other three assessment protocols address the deployment of specific IC services during the period 2017–2018, namely: i) Home hospitalization [13]; ii) Prehabilitation of candidates for major surgery [14]; and, iii) Community-based advanced care service for frail elderly [15, 16]. The ultimate aim of the research is to explore the application of innovative evaluation strategies [4] for IC services deployed in real-life settings. To this end, a comprehensive evaluation of outcomes following a Quadruple Aim approach [17, 18], deployment strategies and maturity of implementation will be performed within each of the four assessment protocols of the study.

The Catalan Health Care System dispenses services for 7.5 inhabitants, providing universal coverage through a tax-based system. Administratively, it is composed by a single public payer and multiple service providers publicly or privately owned. Since 2006, the implementation of IC services in one of the four healthcare sectors in the city of Barcelona (520 k inhabitants) was instituted by the Hospital Clinic of Barcelona (HCB), a tertiary university hospital [19], adopting the Chronic Care Model as the conceptual reference [20, 21]. Moreover, the subsequent Health Plans for Catalonia after 2011, have addressed the deployment challenges by giving priority to new modalities of healthcare delivery for chronic patient care including empowerment of patients and carers. To date, clear examples of clinical effectiveness have been produced for the three IC services presented in this report: Home hospitalization [13, 22, 23], prehabilitation [14] and community-based services for frail patients [9]. It is expected that lessons learned from the implementation of the four protocols reported in the current manuscript will foster regional scalability and sustainability of IC services in Catalonia. Moreover, it is also expected that the recommendations generated by these deployments in real-life settings will significantly contribute to facilitate transferability and comparability of IC services at international level. The context in which these four assessment protocols will take place is described in [11] and [19].

## Methods

The four protocols (Table 1) follow observational and experimental non-randomized study designs. In all cases, comparability between the intervention group and the control group will be achieved using a propensity score matching (PSM) [24, 25] method, as described in detail below. The common methodology for assessing health-value generation of the interventions in each protocol will follow a Quadruple Aim approach [17, 18] considering pre-defined variables for: i) Health and well-being; ii) Experience with care; iii) Operational costs; and, iv) Health professionals’ engagement, as summarized in the second column of Table 2. It is of note that the outcomes of the three first dimensions (Triple Aim approach) [26, 27, 30] will be assessed both separately and jointly. The later will consist of a multi-criteria decision analysis (MCDA) recently developed [31, 32] and currently applied in 17 selected IC programs from 8 European countries [33]. The MCDA approach broadens the scope of the evaluation taking into account patient health reported outcomes and stakeholders’ views on those same outcomes allowing standardized comparisons between seemingly dissimilar IC programmes. Moreover, engagement of health professionals, the fourth pillar of the Quadruple Aim approach, will be assessed using the questionnaires currently applied in [34], aiming at assessing main drivers of large-scale deployment of IC services in 5 European regions.

**Table 1:** Main characteristics of the four assessment protocols

Protocol	Aims	Study design & Measurements	Intervention group	Comparator group	Expected outputs
(1) Population-based study	(1.1) Impact of integrated care on cost-effectiveness	(1.1) Case control study matching registry data using PSM methods (2011–2017) (Additional file 1: Table S1)	(1.1 and 1.2) Residents living in the healthcare district of Barcelona-Esquerra (*n* = 516 K inhabitants)	(1.1 and 1.2) Residents living in the other 3 healthcare districts of Barcelona (~ 400 k inhabitants each), as well as the entire region of Catalonia (*n* = 7.5 M inhabitants)	(1.1a) Health value generation of integrated care
	(1.2) Enhanced health risk assessment and service selection	(1.2) Fixed cohort study			(1.1b) Enhanced Key Performance Indicators (KPI) for long-term assessment of integrated care
					(1.2) Proposal for health risk assessment for service selection
(2) Home hospitalization	(2.1) Assessment of hospital avoidance and early hospital discharge at district level	(2.1) Prospective controlled cohort study using PSM methods (2017–2018) (Additional file 2: Table S2)	(2.1) All patients admitted to the home hospitalization directly from the emergency room (*n* = 800 patients). Study of a deeply characterized subset (triple aim approach) of 200 patients. This subset will be used to generate (2.2).	(2.1) Patients admitted to conventional hospitalization directly from the emergency department of the same hospital (n = 800 patients). Study of a deeply characterized subset (triple aim approach) of 200 patients. This subset will be used to generate (2.2).	(2.1a) Health value generation of the service; expanded HDA using MCDA (n = 200). Factors modulating success of the implementation strategy.
		(2.2) Observational mixed-methods study combining network and cluster analyses with qualitative methodologies			
	(2.2) Recommendations for shared-care agreements between specialized and community-based care				
					(2.1b) KPI for service assessment
					(2.2) Strategies for enhanced interactions between specialized-community-based care.
(3) Prehabilitation	(3.1) Sustainability (cost-effectiveness of prehabilitation at HCB	(3.1) Prospective controlled cohort study using PSM methods (2016–2018) (Additional file 3: Table S3)	(3.1) All candidates for major surgery at HCB receiving prehabilitation (*n* = 500)	(3.1) Candidates for major surgery at HCB receiving usual care in the same hospital (*n* = 250)	(3.1a) Health value generation of prehabilitation at HCB
	(3.2) Recommendations for transition toward a regional peri-operative care program	(3.2) Randomized controlled trial to assess peri-operative care	(3.2) Candidates for major surgery at HCB receiving peri-operative care (*n* = 60)		(3.1b) KPI for service assessment
	(3.3) Enhanced pre-operative risk assessment	(3.3) Fixed cohort study	(3.3) All surgical patients in the last 5 years at HCB	(3.2) Candidates for major surgery at HCB receiving usual care (n = 60)	
					(3.2) Cost-effectiveness of peri-operative care and strategies for regional deployment.
					(3.3) Risk assessment tool for personalized prehabilitation
(4) Frail elderly patients	(4.1) Assessment of community-based integrated care services for frail patients at BSA	(4.1) Prospective controlled cohort study using PSM methods (2018) (Additional file 4: Table S4)	(4.1) Individuals enrolled in BSA integrated care programs for frail elderly that includes: i) Early Discharge support (*n* = 144); ii) Long-term home-based support services (*n* = 566) and iii) Geriatric residences care (*n* = 920)	(4.1) Individuals living in Badalona receiving usual care: i) After hospital discharge (n = 144), ii) At home (n = 566); and, iii) Living at geriatric residences (*n* = 920)	(4.1a) Cost-effectiveness of the service; and, expanded HDA using MCDA (n = 250). Factors modulating success of the implementation strategy.
					(4.1b) KPI for service assessment

**Abbreviations**: *HDA* Health Delivery Assessment, *MCDA* Multi-Criteria Decision Analysis, *HCB* Hospital Clinic de Barcelona, *PSM* Propensity Score Matching, *KPI* Key Performance Indicators for service long-term assessment after the deployment phase, *BSA* Badalona Serveis Asssistencials

**Table 2:** Three main assessment dimensions: effects of the intervention, determinants of success of implementation and maturity of integration

Study Protocol	Outcomes of the intervention [26, 27]		Deployment strategies [28, 29]	Maturity level [4]
(1) Population-based	Mortality, general practitioner visits, community-nurse visits, cumulative days per year admitted in hospital, emergency department visits, all hospital admissions, potentially avoidable hospitalizations, multiple drug prescription, needs for social support, costs per patient per year (Additional file 1: Table S1)		A. What are the possible factors and agents responsible for good implementation of a health intervention? B. What are the possible factors for enhancing or expanding a given health intervention? C. What describes the context in which implementation occurs? D. What describes the main factors influencing implementation in a given context? To be assessed using a mixed methods approach: combining qualitative and quantitative methods	Assessment of the twelve dimensions of the Maturity Model for Integrated Care, both at health system and health services levels, promoted by the European Innovation Partnership for Active and Healthy Ageing, following the instructions reported in reference (4). These twelve dimensions are: 1. Readiness to Change 2. Structure & Governance 3. Information & eHealth Services 4.Standardization& Simplification 5. Finance & Funding 6. Removal of Inhibitors 7. Population Approach 8. Citizen Empowerment 9. Evaluation Methods 10. Breadth of Ambition 11. Innovation Management 12. Capacity Building
(2) Home hospitalization	Health and well-being	Mortality rate 30/90 days after discharge, place of death, avoidable hospital admissions, total bed days, 12 months before admission (hospital and community resources); 30-day after discharge (hospital and community resources), transitional care strategies (palliative care, primary care or hospital care)		
	Patient experience	Person centeredness, continuity of care (Additional file 2: Table S2)		
	Costs	Operational costs		
(3) Prehabilitation	Health and well-being	Cumulative hospital days of stay, intensive care unit length of stay, number of complications per patient, costs from the perspective of the hospital including inpatient services, diagnostic procedures, pharmaceutical consumption and blood products consumption, aerobic capacity, physical activity, psychological status, health status (Additional file 3: Table S3)		
	Costs	Operational costs		
(4) Frail elderly	Health and well-being	Mortality rate, avoidable hospital admissions, total bed days, 30-day readmissions, number of ER visits in the month, physical functioning, psychological well-being, social relationships & participation, enjoyment of life, resilience, autonomy		
	Patient experience	Person centeredness, continuity of care, burden of medication, burden of informal caregiving (Additional file 4: Table S4)		
	Costs	Operational costs		

The current assessment protocols also aim to separately establish key factors that modulate the success of IC service deployments in order to identify their potential for transferability to other sites. To this end, we will use standard implementation science tools [28, 29, 35, 36] to answer the questions delineated in the third column of Table 2, as well as to report the results of the implementation process following standards for reporting implementation studies (StaRI) [28]. This will allow us to identify facilitators, barriers, solutions and critical success factors during the course of the implementation process with relevant implications for analysis of service transferability. It must be highlighted that collaborative tools and methodologies were applied for the implementation of the three service-oriented studies. The process incorporates co-design elements, with participation of different stakeholders, including patients, following a Plan-Do-Study-Act (PDSA) iterative cycles approach [37] adapted to the characteristics of each assessment protocol, as summarized below. Last, but not least, the maturity of the ecosystem in which the service is being deployed will be assessed following the twelve-dimension measurement protocol described in [4] and summarized in the fourth column of Table 2.

It is assumed that the three assessment categories depicted in Fig. 1 and in Table 2: i) Outcomes, ii) Deployment strategies, and, iii) Maturity level, will provide the basis for identification of general, and service-specific, key performance indicators (KPI) useful for long-term follow-up of IC services after the initial deployment period, taking into account outcomes, processes and structure [38].

The assessment protocols will combine three different data sources. First, registry data obtained from the Catalan Health Surveillance System (CHSS) [16, 39, 40], as briefly described below. Second, individual data extracted from the electronic healthcare records from primary care and specialized care. Third, data derived from prospectively applied standardized questionnaires to patients, health professionals and managers (Additional file 1: Table S1, Additional file 2: Table S2, Additional file 3: Table S3 and Additional file 4: Table S4). The challenges involved in the combination of different datasets used in these four assessment protocols have been overcome within the framework of the recent EU General Data Protection Regulation (GDPR) [41].

The CHSS includes updated registries from primary care, hospital-related events (e.g. hospitalization, emergency room and specialized outpatient visits), pharmacy, mental health, socio-sanitary services, respiratory therapies, dialysis, outpatient rehabilitation and non-urgent transport of all citizens living in Catalonia (7.5 M) since 2011. The information is updated every 6 months. It provides a basis for cost analyses of the use of healthcare resources, pharmacy consumption, and prevalence of key health problems. The CHSS feeds the regional population-based risk stratification tool named Adjusted Morbidity Groups (GMA) that complies with the following characteristics: i) A population health approach; ii) No licensing constraints; iii) Open source computational algorithms; and, iv) The adjusted morbidity grouper relies mostly on statistical criteria, as opposed to other tools that include expert-based coefficients, thus facilitating quick transferability to other territories [39, 42].

### Assessment protocols

#### Assessment protocol 1: population-based analysis

**Fig. 2:**
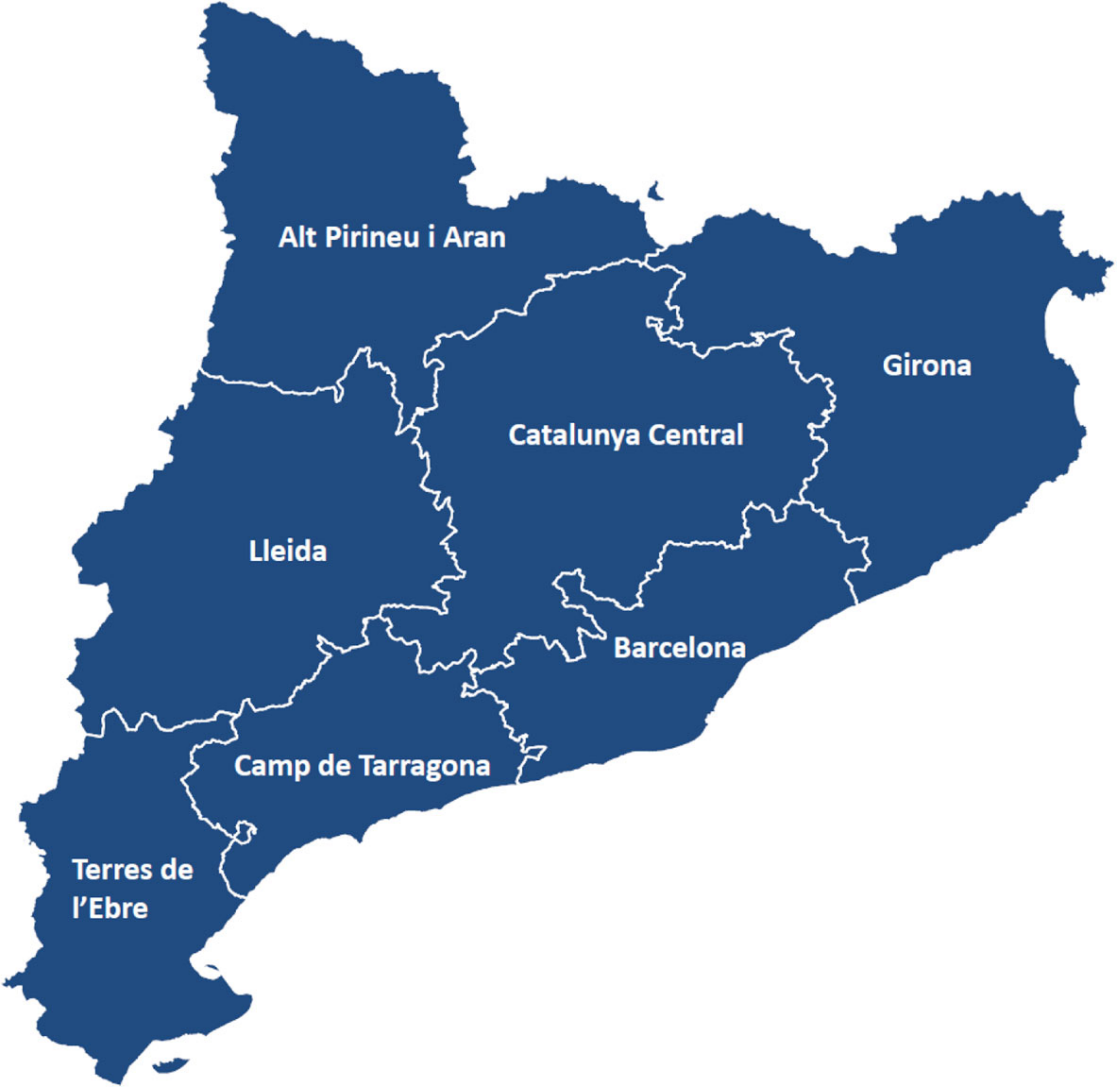
The figure displays the seven health regions of Catalonia. The urban area of Barcelona (1.8 million citizens) has four health districts. The South-Eastern healthcare sector of the Barcelona city, which encompasses 520 k inhabitants, is Barcelona-Esquerra (AISBE). Taken from the Catalan Health Service (CatSalut) website. https://catsalut.gencat.cat/ca/coneix-catsalut/transparencia/territori/informacio-cartografica/mapes/ This is a public access image.

This protocol will take into consideration the entire population of healthcare users in Catalonia. The health system in Catalonia (7.5 M inhabitants) has three organisational levels, with the seven health regions at the top level (Fig. 2). Each region includes several geographical areas called health districts, second level, covering both specialised and primary care needs of the population. The third level corresponds to clusters of primary care centres within each healthcare district. The region has a total of 369 primary care units covering approximately 20 k citizens, on average, each of them.

Integration of health and social services in the entire Catalonia is being promoted under the umbrella of the five-year regional health plans. Key goals in terms of deployment of the integrated model were established during the 2011–2015 Plan [11] and consolidation of the program is expected during the 2016–2020 period [12].

The Integrated Health District in Barcelona-Esquerra (AISBE) (*n* = 520 k inhabitants) [19] is the intervention district and includes HCB as reference centre, two general hospitals and 19 primary care centres run by different healthcare providers. Since mid-2000s, AISBE has deployed, and continuously developed, IC services for chronic patients across healthcare tiers [9, 19]. Deployment of IC services in AISBE is based on the hypothesis that an appropriate transfer of selected care complexities from hospital-based to community-based care, within an IC scenario, can increase healthcare value generation both at provider and at health system levels. The main characteristics and achievements of technologically-supported IC services evaluated and adopted in AISBE have been reported elsewhere [8, 9, 14, 16, 19, 43].

The main objective of this assessment protocol is the analysis of health-value generation of IC in Catalonia (Table 1). An ancillary aim is to enhance health risk assessment for clinical purposes and service selection, taking into account the population-based risk assessment tool, (i.e. GMA), as reported in [39]. For the principal objective, health-related outcomes in AISBE will be compared using a case-control design with three other healthcare districts of the city of Barcelona (approximately 400 k inhabitants each), and the entire region (7.5 M inhabitants), considered as control areas. A PSM method will used for comparability purposes using age, sex, health-risk grading based on GMA [39, 42], and socioeconomic status as matching variables*.* Comparisons between intervention and controls will be done on a yearly basis for the period 2011–2017. Key specific aspects of the assessment protocol are summarized in Additional file 1: Table S1.

Health risk assessment and service selection will address enrichment of the predictive role of standard clinical information using population-based health risk assessment (GMA grading) and patient self-tracked information obtained through the regional personal health folder in Catalonia (La Meva Salut). Evaluation of resulting clinical predictive modelling (Table 1) will be based on fixed cohort study designs with 1 year follow-up, as already reported in [40].

#### Assessment protocol 2: home hospitalization (HH)

The intervention group to be analysed will include all the patients admitted to HH and early discharge service from HCB during a one-year period (October 2017–October 2018) (*n* = 1146), approximately 70% of the patients were admitted to HH directly from the emergency room. A subset of the patients admitted to HH directly from the emergency room throughout the study period will be assessed separately (*n* = 200).

The characteristics of the intervention have recently been described by Hernandez et al. [13] in terms of implementation strategy, outcomes and costs during the deployment of the service in a real-life setting during the years 2006–2015. During the period 2017–2018, the programme was expanded to 48 beds per day to cover the entire AISBE health district.

The principal objective of this protocol is to assess hospital avoidance and early hospital discharge at health district level. Moreover, the approach aims to generate recommendations for shared-care agreements between specialized and community-based care after discharge to ensure safe transitional care strategies.

The assessment protocol will consist of a prospective controlled cohort study wherein patients admitted to HH directly from the emergency room (intervention) (*n* = 800) will be compared with conventional hospitalisation (control) (*n* = 800). The control group will include patients admitted to conventional hospitalization directly from the emergency room of the same hospital (HCB). PSM will be used for comparability purposes using age, sex, GMA, socioeconomic status, number of hospitalisations during the previous year and polypharmacy as matching variables. As described above, a sub-group of 200 consecutive patients recruited on a voluntary basis, admitted through the emergency department during the study period, from each arm (HH and conventional hospitalization) will be also thoroughly characterized using a set of standardized questionnaires [26, 27, 30], as depicted in Tables 2 and Additional file 2: Table S2. It is of note that these two well defined sub-groups of 200 patients each (*n* = 400) will also constitute a single fixed cohort for later analysis on the interactions between specialized and community-based care using network and cluster analyses alongside qualitative methodologies.

#### Assessment protocol 3: Prehabilitation service

This is a preventive intervention targeted at high risk candidates for major surgical procedures carried out preoperatively aiming at reducing complications and enhancing postoperative recovery. It combines: i) Motivational interviewing; ii) High-intensity endurance exercise training; iii) Promotion of physical activity; iv) Nutritional supplementation; and; v) Psychological support.

The intervention is currently deployed as a mainstream service at HCB in several types of major surgeries. During fall 2017, three multidisciplinary workshops using a design-thinking approach were carried out to refine the service workflow and to explore the potential for service scalability. The outcomes of the co-design process provided a robust background for the design of a future personalized perioperative care service at regional level covering three phases: prehabilitation, in-patient care, and post-discharge rehabilitation.

The current assessment protocol aims to assess cost-effectiveness of prehabilitation as a mainstream service in the ongoing deployment at HCB, as well as to generate a roadmap for regional scalability of the service. It is planned as a prospective controlled cohort study including 500 consecutive patients undertaking prehabilitation, as the intervention group, and patients following standard care before surgery, in the same hospital (i.e. HCB), as the control group (2:1 intervention to control ratio). The patients will be included from the following type of surgeries: major digestive surgery (*n* = 525), lung volume reduction (*n* = 30), radical cystectomy (*n* = 30), major cardiovascular surgery (*n* = 165). Study groups will be made comparable using PSM with the following matching variables: type of surgery, age, sex, American Society of Anaesthesiologists index and GMA grading. Patients’ clinical outcomes will be assessed at baseline, pre-surgery and 30 days after surgery. The primary outcome will be cost-effectiveness, meaning reduced hospital stay and early re-admissions. Secondary outcome variables will include number of complications per patient, healthcare use, aerobic capacity, physical activity and psychological and health status. The specificities of the assessment protocol are summarized in Additional file 3: Table S3.

#### Assessment protocol 4: community-based care for the frail elderly

The assessment protocol will evaluate three types of specific interventions during the period from 1st January to 31th December 2018: i) Early discharge service (*n* = 144) which includes acute patients admitted to the medical and/or surgical hospital wards and promptly discharged to receive home-based post-acute care and/or rehabilitation; ii) Home-based Case Management service (*n* = 566) which includes complex chronic patients or patients receiving long-term care by a case management nurse; and, iii) Geriatric residences service (*n* = 920) will include patients receiving acute support, post-acute or continued care for elderly people living in geriatric residences. It will be conducted by Badalona Serveis Assistencials (BSA), an IC service provider located in the city of Badalona (216 K inhabitants) in the North-Eastern part of the Barcelona Metropolitan Area.

The current assessment protocol, summarized in Additional file 4: Table S4, aims to assess cost-effectiveness of these three interventions for frail patients, as well as to generate a roadmap for regional scalability of the service. The study protocol will consist of a prospective controlled cohort study wherein each intervention group will be compared with the corresponding usual care group (controls, 1:1 ratio) (*n* = 1630 in each arm), using propensity score matching. Age, sex, GMA, socioeconomic status, number of hospitalisations during the previous year and polypharmacy will be used as matching variables. The patients from the usual care group will be recruited during the study period in the same area. A subset of 250 patients from each control and intervention groups will be thoroughly characterized using a set of standardized questionnaires [26, 27, 30], as depicted in Additional file 4; Table S4.

### Additional elements toward enhancement of IC services

All four assessment protocols will also integrate the following dimensions described below.

#### Enhanced risk assessment & service selection

The 2011–2015 Catalan Health Plan extensively implemented a case finding system classifying high risk chronic patients into two different categories based on defined criteria and primary care physician judgement: i) Complex chronic patients (CCP, approximately 3% of the population); and, ii) Patients with less than 12 months expected life survival (Advanced Care Disease, ACD, approximately 1% of the population). The latter category of patients consists of citizens with advanced chronic diseases and/or with oncological problems being potential candidates for palliative care.

Since 2015, the population-based risk stratification tool (i.e. GMA) primarily used for health policy purposes, has been extensively implemented in primary care. The clinical workstation currently displays the GMA grading of the patient being attended by the health professionals, without specific connections with the patient’s care plan. The current assessment protocols offer an opportunity to explore enhanced clinical risk assessment modalities aiming at facilitating preventive strategies, improving service selection and providing clinical decision support. To this end, the assessment protocols will elaborate and evaluate novel approaches to health risk assessment following the orientations described in [39, 40, 42].

#### Assessment of technological support

The three service-oriented assessment protocols will assess acceptability, usability and value generation of digital tools supporting the different services with focus on personal health systems, and collaborative adaptive case management (ACM). Since these key supporting technologies are required to be integrated with provider-specific and regional health information systems for a large-scale implementation in the region (i.e., Catalonia), the protocols will be built upon the regional digital health framework [44] (Additional file 5: Figure S1). Specifically, two personal health systems for patient self-management at community level are being tested: i) MyPathway® (http://mypathway.healthcare); and, ii) CONNECARE Self-Management System (SMS) [45]. The former is a secure digital communications channel connecting patients to clinicians and services. It is a browser and app-based commercial application to use on phones, tablets and PCs. The SMS is a prototype application to use on smartphones that allow patients’ self-tracking, monitoring by health care professionals and bi-directional messaging to improve the patients’ treatment and encourage them in following it.

The assessment protocols also consider ACM as key supporting technology [46–48] to enhance collaborative work among health professionals and patients themselves (actively participating in his/her healthcare via the above personal health systems). To this end, an ACM process based on the Camunda® open-source platform (https://camunda.org) was selected to support process workflow specification, case management and decision automation. The ACM process engine is aimed at providing the required process engine functionality to current hospital information systems.

Acceptability (by means of 3 Likert scales alongside a net promoter score) [49] and usability (by means of the System Usability Scale - SUS) [50] of MyPathway® and/or SMS will be assessed by patients (at patient discharge from the protocols), and of ACM process engine (i.e. Camunda®) by healthcare professionals. Moreover, assessment of consolidated implementation of the digital health tools supporting each of the four assessment protocols will be done using the mini-MAST tool [51] (Additional file 6: Annex S1).

#### Co-design activities

Deployment of the Catalan Health Plans involves a highly structured co-design system ensuring follow-up and continuous improvement of the different implementation initiatives. Likewise, the deployment of IC within AISBE has a well-defined structure of committees at different levels ensuring refinement of the implementation processes, as described in detail in [19]. Moreover, two of the EU projects supporting the current assessment protocols [34, 45] have built-in co-design protocols applying collaborative tools and methodologies following a PDSA (Plan, Do, Study, Act) approach [37]. The PDSA cycles are a systematic series of steps for gaining valuable learning and knowledge for the continual improvement of a product or process. All in all, the different levels of co-design activities alluded to above provide information for undertaking a mixed-methods approach combining quantitative and qualitative methodologies to assess implementation of IC services, as indicated Table 2, third column.

## Discussion

The current document provides the core information on a framework applicable for the evaluation of large-scale deployment of IC services in Catalonia. The approach relies on the use of assessment of shared interventions, within well-defined service workflows, that have been previously tested in terms of efficacy and potential for value generation. The three assessment categories depicted in Table 2: i) Value generation of IC services following standard and novel approaches, i.e. MCDA; ii) Deployment strategies; and, iii) Maturity level of the ecosystem for implementation will provide the basis for a comprehensive evaluation of IC and should contribute to the identification of KPIs useful for long-term follow-up after IC service adoption (Fig. 1).

Observational and experimental non-randomized controlled cohort study designs using PSM have been adopted, instead of randomized controlled trials, as a pragmatic option to assess events in a real-life setting [52, 53] The assessment protocols also take into account the role of digital health as enabling tools supporting different strategic aspects of care coordination, namely: service scalability, service evaluation and personalization through enhanced service selection, as described in [39].

We believe that the current regional context in Catalonia facilitates full alignment between the Catalan Health Plan 2016–2020 [12] and the ongoing Nextcare program [54] aiming at fostering innovation of digitally-supported healthcare services for chronic patients with multimorbid conditions. It is of note that Nextcare acts as an umbrella program wherein three EU projects with similar timeframes converge covering complementary facets of IC implementation, namely: i) CONNECARE [45], addressing enhanced digital support of IC services; ii) SELFIE [33], exploring novel modalities of health delivery assessment like multi-criteria decision analysis; and, iii) ACT@Scale [34], analysing key factors that modulate large scale deployment of IC services. All in all, the scenario described facilitates the progressive expansion of the results of the assessment protocols to analyses of other IC services (i.e. non-invasive home-based ventilation, cardiopulmonary rehabilitation of chronic patients, etc.) and to distinct healthcare districts toward achievement of effective full regional deployment of care coordination.

Real-life assessment of IC services using the proposed implementation research methodologies will contribute to quantify health value generation of care coordination. The approach should also contribute to generating recommendations for transferability of the services facilitating outcomes comparability across sites.

## Additional files

Additional file 1: **Table S1**. Population-based protocol. (DOCX 26 kb)

Additional file 2: **Table S2**. Home Hospitalization protocol. (DOCX 35 kb)

Additional file 3: **Table S3**. Prehabilitation protocol. (DOCX 28 kb)

Additional file 4: **Table S4**. Three interventions addressed to frail complex chronic patients. (DOCX 35 kb)

Additional file 5: **Figure S1**- Digital health framework in Catalonia (IS3). (DOCX 75 kb)

Additional file 6: **Annex 1** - Method for ASsessment of Telemedicine (mini-MAST). (DOCX 22 kb)

## Data Availability

Not applicable.

## Abbreviations

ACD: Advanced care disease
ACM: Adaptive case management
AISBE: The Integrated Health District in Barcelona-Esquerra
BSA: Badalona Serveis Assistencials
CCP: Complex chronic patients
CHSS: Catalan Health Surveillance System
GDPR: General data protection regulation
GMA: Adjusted morbidity groups
HCB: Hospital Clinic of Barcelona
HDA: Health delivery assesment
IC: integrated care
KPI: Key performance indicator
MCDA: multi-criteria decision analysis
PDSA: Plan-Do-Study-Act
PSM: Propensity score matching
SMS: Self-managemet system
StaRI: Standards for reporting implementation studies

## References

[1] Spreeuwenberg C, Kodner DL (2002). Integrated care: meaning, logic, appplications and implications - a discussion paper. Int J Integr Care.

[2] Lewis R, Rosen R, Godwin N, Dixon J (2010). Where next for integrated care organisations in the English NHS?.

[3] Goodwin N, Alonso A, Meyer I, Muller S, Kubitschke L (2014). Understanding integrated care: the role of information and communication technology. Achieving effective integrated e-care beyond the silos. IGI Global.

[4] Expert Group on Health Systems Performance Assessment. Blocks - tools and methodologies to assess integrated care in Europe. 2017. Available from: https://ec.europa.eu/health/sites/health/files/systems_performance_assessment/docs/2017_blocks_en_0.pdf%0Ahttp://www.journals.cambridge.org/abstract_S1041610215000137%0Ahttp://www.euro.who.int/__data/assets/pdf_file/0005/322475/Integrated-care-models-over

[5] Araujo de Carvalho I, Epping-Jordan J, Pot AM, Kelley E, Toro N, Thiyagarajan JA (2017). Organizing integrated health-care services to meet older people’s needs. Bull World Health Organ.

[6] European Innovation Partnership for Active and Healthy Ageing (EIP-AHA). Available from: https://ec.europa.eu/eip/ageing/home_en

[7] Busse R, Stahl J (2014). Integrated care experiences and outcomes in Germany, the Netherlands, and England. Health Aff.

[8] Cano I, Alonso A, Hernandez C, Burgos F, Barberan A, Roldan J (2015). An adaptive case management system to support integrated care services: lessons learned from the NEXES project. J Biomed Inform.

[9] Hernandez C, Alonso A, Garcia-Aymerich J, Grimsmo A, Vontetsianos T, Cuyàs FG (2015). Integrated care services: lessons learned from the deployment of the NEXES project. Int J Integr Care.

[10] Dates M, Lennox-chhugani N, Sant H, Pereira A, Tedeschi M. Health system performance assessment – integrated care assessment ( 20157303 HSPA). European union; 2018.

[11] Government of Catalonia M of H. Health plan for Catalonia 2011-2015. 2012. Available from: http://www.govern.cat/pres_gov/AppJava/docrel/nota-premsa/contingut/download/89293.htm.

[12] Department of Health. Catalonia health plan for 2016–2020 (in Catalan). 2016. Available from: http://salutweb.gencat.cat/ca/el_departament/Pla_salut/pla-de-salut-2016-2020/.

[13] Hernandez C, Garcia-Aymerich J, Alonso A, Grimsmo A, Vontetsianos T, Garcia-Cuyas F (2018). Implementation of home hospitalization and early discharge as an integrated care service: a ten years pragmatic assessment. Int J Integr Care.

[14] Barberan-Garcia A, Ubré M, Roca J, Lacy AM, Burgos F, Risco R (2018). Personalised Prehabilitation in high-risk patients undergoing elective major abdominal surgery : a randomized blinded controlled trial. Ann Surg.

[15] Hernandez C, Aibar J, De Batlle J, Gomez-Cabrero D, Soler N, Duran-Tauleria E (2015). Assessment of health status and program performance in patients on long-term oxygen therapy. Respir Med.

[16] Cano I, Dueñas-Espín I, Hernandez C, De Batlle J, Benavent J, Contel JC (2017). Protocol for regional implementation of community-based collaborative Management of Complex Chronic Patients. npj Prim Care Respir Med.

[17] Bodenheimer T, Sinsky C (2014). From triple to quadruple aim: Care of the Patient Requires Care of the provider. Ann Fam Med.

[18] West CP (2016). Physician well-being: expanding the triple aim. J Gen Intern Med.

[19] Font D, Escarrabill J, Gómez M, Ruiz R, Enfedaque B, Altimiras X (2016). Integrated health care Barcelona Esquerra (Ais-be): a global view of Organisational development, re-engineering of processes and improvement of the information systems. The role of the Tertiary University hospital in the transformation. Int J Integr Care.

[20] Wagner EH, Austin BT, Von Korff M (1996). Organizing care for patients with chronic illness. Milbank Q..

[21] Wagner EH, Austin BT, Davis C, Hindmarsh M, Schaefer J, Bonomi A (2001). Improving chronic illness care: translating evidence into action. Health Aff.

[22] Hernandez C, Casas A, Escarrabill J, Alonso J, Puig-Junoy J, Farrero E (2003). Home hospitalisation of exacerbated chronic obstructive pulmonary disease patients. Eur Respir J.

[23] Casas A, Troosters T, Garcia-Aymerich J, Roca J, Hernandez C, Alonso A (2006). Integrated care prevents hospitalisations for exacerbations in COPD patients. Eur Respir J.

[24] Austin PC (2011). An introduction to propensity score methods for reducing the effects of confounding in observational studies. Multivariate Behav Res.

[25] Brookhart MA, Schneeweiss S, Rothman KJ, Glynn RJ, Avorn J, Stürmer T (2006). Variable selection for propensity score models. Am J Epidemiol.

[26] Stiefel M, Nolan K. A guide to measuring the triple aim: population health, experience of care, and per capita cost. IHI innovation series white paper. Cambridge, Massachusetts: Institute for Healthcare Improvement; 2012.

[27] Berwick DM, Nolan TW, Whittington J (2008). The triple aim: care, health, and cost. Health Aff.

[28] Pinnock H, Barwick M, Carpenter CR, Eldridge S, Grandes G, Griffiths CJ (2017). Standards for reporting implementation studies (StaRI): explanation and elaboration document. BMJ Open.

[29] Peters DH, Adam T, Alonge O, Agyepong IA, Tran N (2013). Implementation research: what it is and how to do it. Br Med J.

[30] Whittington JW, Nolan K, Lewis N, Torres T (2015). Pursuing the triple aim: the first 7 years. Milbank Q.

[31] Tsiachristas A, Cramm JM, Nieboer A, Rutten- van Mölken M (2013). Broader economic evaluation of disease management programs using multi-criteria decision analysis. Int J Technol Assess Health Care.

[32] Rutten-Van Mölken M, Leijten F, Hoedemakers M, Tsiachristas A, Verbeek N, Karimi M (2018). Strengthening the evidence-base of integrated care for people with multi-morbidity in Europe using multi-criteria decision analysis (MCDA). BMC Health Serv Res.

[33] SELFIE (2016–19) – sustainable integrated care models for multimorbidity delivery, financing and performance. Available from: https://www.selfie2020.eu/

[34] ACT@Scale (2016–19) – Advancing Care Coordination and Telehealth at Scale [Internet]. Available from: https://www.act-at-scale.eu/

[35] Warner G, Lawson B, Sampalli T, Burge F, Gibson R, Wood S (2018). Applying the consolidated framework for implementation research to identify barriers affecting implementation of an online frailty tool into primary health care: a qualitative study. BMC Health Serv Res.

[36] Kirk MA, Kelley C, Yankey N, Birken SA, Abadie B, Damschroder L (2016). A systematic review of the use of the consolidated framework for implementation research. Implement Sci.

[37] Taylor MJ, McNicholas C, Nicolay C, Darzi A, Bell D, Reed JE (2014). Systematic review of the application of the plan-do-study-act method to improve quality in healthcare. BMJ Qual Saf.

[38] Donabedian A (1988). The quality of care: how can it be assessed?. JAMA..

[39] Dueñas-Espín I, Vela E, Pauws S, Bescos C, Cano I, Cleries M (2016). Proposals for enhanced health risk assessment and stratification in an integrated care scenario. BMJ Open.

[40] Vela E, Tényi Á, Cano I, Monterde D, Cleries M, Garcia-Altes A (2018). Population-based analysis of patients with COPD in Catalonia: a cohort study with implications for clinical management. BMJ Open.

[41] European General Data Protection Regulation (GDPR) [Internet]. 2018. Available from: https://eur-lex.europa.eu/legal-content/EN/TXT/PDF/?uri=CELEX:32016R0679&fro

[42] Monterde D, Vela E, Clèries M, grupo colaborativo GMA (2016). Los grupos de morbilidad ajustados: nuevo agrupador de morbilidad poblacional de utilidad en el ámbito de la atención primaria. Atención Primaria.

[43] Barberan-Garcia A, Vogiatzis I, Solberg HS, Vilaró J, Rodríguez DA, Garåsen HM (2014). Effects and barriers to deployment of telehealth wellness programs for chronic patients across 3 European countries. Respir Med.

[44] Modol JR, Aanestad M, Grisot M, Hanseth O, Vassilakopoulou P (2017). Navigating Towards Self-Care: The Catalan Public Patient Portal. Information infrastructures within European health care: working with the Installed Base.

[45] CONNECARE (2016-2019) – Personalized Connected Care for Complex Chronic Patients. Available from: http://www.connecare.eu/

[46] Ardzevičiūte D, Skersys T KE. Evaluation of business process management systems. CEUR workshop proceedings. 2017. Available from: https://hrcak.srce.hr/file/237864

[47] Swenson K. Mastering the Unpredictable Vol. XXXIII, Uma ética para quantos? 2012. p. 81–7.

[48] Herrmann C, Kurz M, Schmidt W (2011). Adaptive case management: supporting knowledge intensive processes with IT systems BT - S-BPM ONE - learning by doing - doing by learning.

[49] Reichheld FF (2003). The one number you need to grow. Harv Bus Rev.

[50] Sauro J. Measuring usability with the system usability scale (SUS). Measuring Usability. 2011. Available from: https://measuringu.com/umux-lite/

[51] Kidholm K, Ekeland AG, Jensen LK, Rasmussen J, Pedersen CD, Bowes A (2012). A model for assessment of telemedicine applications: mast. Int J Technol Assess Health Care.

[52] Institute of Medicine. Integrating research and practice: health system leaders working toward high-value care: workshop summary. Alper J, Grossmann C, editors. Washington, DC: the National Academies Press; 2015. 25834870

[53] Gershon AS, Jafarzadeh SR, Wilson KC, Walkey AJ (2018). Clinical knowledge from observational studies: everything you wanted to know but were afraid to ask. Am J Respir Crit Care Med.

[54] Nextcare. Innovation in integrated Care Services for Chronic Patients, COMRDI15-1-0016. 2016. Available from: http://www.nextcarecat.cat/

