# Supplementary material for: Evaluation of integrated care services in Catalonia: population-based and service-based real-life deployment protocols
Source: BMC Health Serv Res. 2019 Jun 11;19:370. doi: 10.1186/s12913-019-4174-2 (PMC6560864; doi:10.1186/s12913-019-4174-2)
Supplement: Supplementary file 1 — Table S1. Population-based protocol. (DOCX 26 kb) [file 12913_2019_4174_MOESM1_ESM.docx]

**Additional file 1: TABLE S1**. **Population-based protocol**

The table shows the detailed proposed evaluation for the population-based protocol according to the elements and dimensions described in the main text.

| **Objective** | | To assess health-care value generation of the implementation of integrated care services within a population health program |
| --- | --- | --- |
| **Study design** | | Case-control study matching registry data using PSM methods. Yearly-based analysis between 2011 and 2017 |
| **Study subjects** | | All adults living within AISBE health district (intervention group) and adult population living in the other three urban health districts in Barcelona and the entire region of Catalonia (control groups) |
| **Inclusion criteria** | | All living adults (>18 years old) residing in Catalonia; having data registered in the CHSS |
| **Exclusion criteria** | | n/a |
| **Variables & measurement tools** | **Health and well-being** | Mortality, general practitioner visits, cumulative days per year admitted in hospital, emergency department visits, hospital admissions, hospitalization-days per year, potentially avoidable hospitalizations. Data will be extracted from the CHSS |
|  | **Patient experience** | Use of citizen-reported outcomes from the ESCA (1) survey to generate input data for a population-based MCDA (2,3) approach |
|  | **Costs analysis** | The following items will be extracted from the CHSS: primary care use, hospitalization, emergency room and specialized outpatient visits, pharmacy, mental health, socio-sanitary services, respiratory therapies, dialysis, outpatient rehabilitation and non-urgent transport. |
|  | **Staff engagement** | Periodic surveys and yearly meetings with professional leadership |
| **Statistical analysis** | | Propensity score matching using age, sex, health-risk scoring based on GMA (4,5), and socioeconomic status |
| **Expected outcomes** | | Recommendation of strategies for contributing to achieve full integrated care coverage at regional level |
| **Health risk assessment** | | Enrich/evolve current case-finding strategies using the current population-based risk assessment tool (GMA) |
| **Digital supporting tools** | | Develop GDPR-compliant strategies for enhanced data analytics of health and social care data from multiple sources |
| **Co-design activities** | | Described in the Catalan Health Plans 2011-2015 and 2016-2020, as well as co-design strategies in AISBE (6) |
| **Future developments** | | Enrich service-based predictive modelling using population-based risk assessment tools (GMA) and patients’ self-tracking data |

AISBE: Integrated Health District in Barcelona-Esquerra; CHSS: Catalan Health Surveillance System; GMA: Adjusted Morbidity Groups, population-based health risk assessment tool; ESCA: Catalan Health Survey; MCDA: Multi-criteria decision analysis: PSM: Propensity Score Matching

**References:**

1. Enquesta de Salut de Catalunya (ESCA). Available from: http://salutweb.gencat.cat/ca/el_departament/estadistiques_sanitaries/enquestes/esca//

2. Tsiachristas A, Cramm JM, Nieboer A, Rutten- van Mölken M. Broader Economic Evaluation Of Disease Management Programs Using Multi-Criteria Decision Analysis. Int J Technol Assess Health Care. 2013;29:301–8.

3. Rutten-Van Mölken M, Leijten F, Hoedemakers M, Tsiachristas A, Verbeek N, Karimi M, et al. Strengthening the evidence-base of integrated care for people with multi-morbidity in Europe using Multi-Criteria Decision Analysis (MCDA). BMC Health Serv Res. 2018;18:576.

4. Monterde D, Vela E, Clèries M, grupo colaborativo GMA. Los grupos de morbilidad ajustados: nuevo agrupador de morbilidad poblacional de utilidad en el ámbito de la atención primaria. Atención Primaria. 2016;48:674–82.

5. Dueñas-Espín I, Vela E, Pauws S, Bescos C, Cano I, Cleries M, et al. Proposals for enhanced health risk assessment and stratification in an integrated care scenario. BMJ Open. 2016;6:e010301.

6. Font D, Escarrabill J, Gómez M, Ruiz R, Enfedaque B, Altimiras X. Integrated Health Care Barcelona Esquerra (Ais-Be): A Global View of Organisational Development, Re-Engineering of Processes and Improvement of the Information Systems. The Role of the Tertiary University Hospital in the Transformation. Int J Integr Care. 2016;16:1–10.
