# Supplementary material for: Evaluation of integrated care services in Catalonia: population-based and service-based real-life deployment protocols
Source: BMC Health Serv Res. 2019 Jun 11;19:370. doi: 10.1186/s12913-019-4174-2 (PMC6560864; doi:10.1186/s12913-019-4174-2)
Supplement: Supplementary file 2 — Table S2. Home Hospitalization protocol. (DOCX 35 kb) [file 12913_2019_4174_MOESM2_ESM.docx]

**Additional file 2:** **TABLE S2.** **Home Hospitalization protocol**

The table shows the detailed proposed evaluation for the Home Hospitalization protocol according to the elements and dimensions described in the main text.

| **Objective** | | 1. To assess value generation of hospital avoidance and early hospital discharge at health district level; 2. To generate recommendations for shared-care agreements between specialized and community-based care after discharge |
| --- | --- | --- |
| **Study design** | | Prospective controlled cohort study design using propensity score matching, of patients admitted directly from the emergency room (n= 800 in each study arm), with a sub-group analysis (n= 200 in each study arm) |
| **Study subjects** | | All the patients admitted to Home Hospitalization and early discharge (October 2017 - October 2018, n=1,146), approximately 70% of them directly admitted to HH from the emergency room (n= 800). A deeply characterized subset of HH patients admitted directly from the emergency room, recruited consecutively on a voluntary basis, will be assessed separately. The control group will consist of conventional hospitalization patients admitted directly from the emergency room of the same hospital. |
| **Inclusion criteria** | | All patients fulfilling criteria for hospital admission; living in his/her house within the healthcare district of AISBE; having carer 24h per day; having phone |
| **Exclusion criteria** | | High risk of severe clinical deterioration not treatable at home, as assessed by best medical judgment; admission in a short stay unit; severe psychiatric disorder; insufficient manpower of the professional team running the program for additional admissions to Home Hospitalization. |
| **Variables & measurement tools** | **Health and well-being** | Mortality rate 30/90 days after discharge, place of death, avoidable hospital admissions, total bed days, 12 months before admission (hospital and community resources); 30-day after discharge (hospital and community resources), transitional care strategies (palliative care, primary care or hospital care); 90 days after discharge (mortality rate)  Hospital resources: admissions (planned and unplanned, Emergency room visits, Home visits, outpatient visits)  Community resources: general practitioner visits; community nurse visits; social worker visits  The sub-group characterization will also include: Physical function (SF-36 (1)); psychological well-being (MHI-5 (2)); social relationships & participation (IPA (3)) ; enjoyment of life (ICECAP-O (4)); resilience (BRS (5)); activation and engagement (PAM-13 (6)) |
|  | **Patient experience** | Person centeredness (P3CEQ (7)); continuity of care (NCQ (8)) (applied for the sub-group characterization only) |
|  | **Costs analysis** | Operational / running costs will be analysed for the following categories: staff, pharmacological and non-pharmacological treatment, consumables, diagnostic tests and procedures and transport. Also, structural costs will be included as a separate category. The analysis period will include 12 months before admission, during hospitalization and 30/90 days after discharge |
|  | **Staff engagement** | Questionnaires for managers and health professionals from (9) |
| **Statistical analysis** | | A - Propensity score matching using age, sex, GMA (10,11), socioeconomic status, number of hospitalisations during the previous year and polypharmacy as matching variables. Health delivery assessment analyses comparing intervention and control groups. Identification of variables with predictive value.   1. Network and cluster analyses alongside qualitative methodologies for the shared-care between specialized and community-based care sub-group analysis. |
| **Expected outcomes** | | Identification of key performance indicators for outcomes (MCDA (12,13) and others), process and structure |
| **Health risk assessment** | | A - Elaborate service-based risk predictive modelling based on historical data from Home Hospitalization and conventional hospitalization addressing mortality and readmission at 30/90 days.  B - Patient stratification according to community services based predictive models derived from sub-group analysis. |
| **Digital supporting tools** | | A - Use of a virtual private network to remotely access hospital information systems. Development of a secure videoconference tool to partially replace face to face home-based visits  B - Use of MyPathway® to support communication between community-based and specialized care. |
| **Co-design activities** | | Iterative PDSA cycles will allow to refine service workflows and digital supporting tools. Each full PDSA cycle will be completed every six months |
| **Future developments** | | A – Refinement of service-based risk predictive modelling tools  B - Expand digital support of care coordination through integration of apps into the regional personal health folder, La Meva Salut. |

AISBE: Integrated Health District in Barcelona-Esquerra; GMA: Adjusted Morbidity Groups, population-based health risk assessment tool; ICECAP-O: ICEpop Capability Measure for Older People Questionnaire; IPA: Impact on Participation and Autonomy Questionnaire; MCDA: Multi-criteria decision analysis;MHI-5: Mental Health Inventory Questionnaire; NCQ: Nijmegen Continuity of Care Questionnaire; P3CEQ; Person Centred Coordinated Experience Questionnaire; PAM-13: Patient Activation Measure questionnaire; PDSA: Plan-Do-Study-Act; SF-36: 36-Item Short form Survey.

**References:**

1. Alonso J, Prieto L, Antó JM. [The Spanish version of the SF-36 Health Survey (the SF-36 health questionnaire): an instrument for measuring clinical results]. Med Clin (Barc). 1995;104:771–6.

2. Brazier JE, Harper R, Jones NM, O’Cathain A, Thomas KJ, Usherwood T, et al. Validating the SF-36 health survey questionnaire: new outcome measure for primary care. BMJ. 1992;305:160–4.

3. Cardol M, de Haan RJ, van den Bos GAM, de Jong BA, de Groot IJM. The development of a handicap assessment questionnaire: the Impact on Participation and Autonomy (IPA). Clin Rehabil. 1999;13:411–9.

4. Coast J, Peters TJ, Natarajan L, Sproston K, Flynn T. An assessment of the construct validity of the descriptive system for the ICECAP capability measure for older people. Qual Life Res. 2008;17:967–76.

5. Smith BW, Dalen J, Wiggins K, Tooley E, Christopher P, Bernard J. The brief resilience scale: Assessing the ability to bounce back. Int J Behav Med. 2008;15:194–200.

6. Hibbard JH, Mahoney ER, Stockard J, Tusler M. Development and Testing of a Short Form of the Patient Activation Measure. Health Serv Res. 2005;40:1918–30.

7. P3CEQ. Person centred coordinated experience questionnaire. Available from: http://p3c.org.uk/prom-detail/29

8. Uijen AA, Schellevis FG, van den Bosch WJHM, Mokkink HGA, van Weel C, Schers HJ. Nijmegen Continuity Questionnaire: Development and testing of a questionnaire that measures continuity of care. J Clin Epidemiol. 2011;64:1391–9.

9. ACT@Scale (2016-19) – Advancing Care Coordination and Telehealth at Scale. Available from: https://www.act-at-scale.eu/

10. Monterde D, Vela E, Clèries M, grupo colaborativo GMA. Los grupos de morbilidad ajustados: nuevo agrupador de morbilidad poblacional de utilidad en el ámbito de la atención primaria. Atención Primaria. 2016;48:674–82.

11. Dueñas-Espín I, Vela E, Pauws S, Bescos C, Cano I, Cleries M, et al. Proposals for enhanced health risk assessment and stratification in an integrated care scenario. BMJ Open. 2016;6:e010301.

12. Tsiachristas A, Cramm JM, Nieboer A, Rutten- van Mölken M. Broader Economic Evaluation Of Disease Management Programs Using Multi-Criteria Decision Analysis. Int J Technol Assess Health Care. 2013;29:301–8.

13. Rutten-Van Mölken M, Leijten F, Hoedemakers M, Tsiachristas A, Verbeek N, Karimi M, et al. Strengthening the evidence-base of integrated care for people with multi-morbidity in Europe using Multi-Criteria Decision Analysis (MCDA). BMC Health Serv Res. 2018;18:576.
