# Supplementary material for: Evaluation of integrated care services in Catalonia: population-based and service-based real-life deployment protocols
Source: BMC Health Serv Res. 2019 Jun 11;19:370. doi: 10.1186/s12913-019-4174-2 (PMC6560864; doi:10.1186/s12913-019-4174-2)
Supplement: Supplementary file 3 — Table S3. Prehabilitation protocol. (DOCX 28 kb) [file 12913_2019_4174_MOESM3_ESM.docx]

**Additional file 3: TABLE S3. Prehabilitation protocol**

The table shows the detailed proposed evaluation for the prehabilitation protocol according to the elements and dimensions described in the main text.

| **Objective** | | 1. To asses cost-effectiveness and long-term sustainability of the service 2. To elaborate a roadmap for regional scale-up of the service |
| --- | --- | --- |
| **Study design** | | Prospective controlled cohort study (n=750 patients) (2:1 ratio) made comparable with a propensity score matching method. |
| **Study subjects** | | Patients participating in the Prehabilitation program at HCB and prospective sample of contemporaneous controls |
| **Inclusion criteria** | | Candidates to colorectal surgery, esophagectomy, gastrectomy, gastric bypass, major liver resection, pancreas resection, lung volume resection, radical cystectomy, cardiac valve surgery or cardiac revascularization at high-risk for surgical complications defined by age > 70 years old and/or ASA 3-4. |
| **Exclusion criteria** | | Non-elective surgery; metastatic disease known preoperatively; unstable cardiac or respiratory disease; locomotor limitations precluding exercise performance; cognitive deterioration impeding adherence to the program |
| **Variables & measurement tools** | **Health and well-being** | Number and severity of complications per patient (daily chart review); health status (SF-36 (1)); psychological status (HAD (2)); physical activity (YPAS (3)); aerobic capacity (6MWT (4)) |
|  | **Patient experience** | Patient satisfaction survey |
|  | **Costs analysis** | Costs from the perspective of the hospital including inpatient services, diagnostic procedures, pharmaceutical consumption and blood products consumption |
|  | **Staff engagement** | Questionnaires for managers and health professionals from (5) |
| **Statistical analysis** | | Propensity score matching using age, sex, ASA, type of surgery and GMA (6,7) as matching variables. Health delivery assessment analyses comparing intervention and control groups. Identification of variables with predictive value. |
| **Expected outcomes** | | Assessment of factors modulating implementation success; identification of KPI for long-term assessment of the service generation of recommendations for service transferability at regional level (Catalonia) |
| **Health risk assessment** | | Enhancement of current risk predictive rules (age and ASA) through elaboration of multilevel (clinical data, GMA score) predictive modelling for clinical decision support |
| **Digital supporting tools** | | MyPathway® app for physical activity prescription, monitoring and patient education interoperable with hospital EMR |
| **Co-design activities** | | Several co-design sessions involving all stakeholders, covering: 1) refinement of service workflow; 2) digital support requirements; and, 3) strategies for regional scalability. Moreover, iterative six-month PDSA cycles will be used to assess service workflow and improve digital supporting tools |
| **Future developments** | | A – Elaboration and assessment of a perioperative care service addressing: i) Personalized prehabilitation; ii) In-patient care preventing postoperative complications; and, iii) Postoperative care to speed-up functional recovery of the patients (RCT including 120 patients).  B - Regional scalability of the prehabilitation program |

HCB: Hospital Clinic de Barcelona; ASA: American Society of Anesthesiologists; SF-36: 36-Item Short form Survey; YPAS: Yale Physical Activity Survey; HAD: Hospital Anxiety and Depression Scale; 6MWT: 6-minute walking test; PAM-13: Patient Activation Measure Questionnaire; GMA: Adjusted Morbidity Groups, population-based health risk assessment tool; KPI: key performance indicators; EMR: electronic medical record; RCT: randomized controlled trial.

**References:**

1. Alonso J, Prieto L, Antó JM. [The Spanish version of the SF-36 Health Survey (the SF-36 health questionnaire): an instrument for measuring clinical results]. Med Clin (Barc). 1995;104:771–6.

2. Zigmond AS, Snaith RP. The Hospital Anxiety and Depression Scale. Acta Psychiatr Scand. 2018;67:361–70.

3. Donaire-Gonzalez D, Gimeno-Santos E, Serra I, Roca J, Balcells E, Rodríguez E, et al. Validation of the Yale Physical Activity Survey in Chronic Obstructive Pulmonary Disease Patients. Arch Bronconeumol. 2011;47:552–60.

4. Holland AE, Spruit MA, Troosters T, Puhan MA, Pepin V, Saey D, et al. An official European respiratory society/American thoracic society technical standard: Field walking tests in chronic respiratory disease. Eur Respir J. 2014;44:1428–46.

5. ACT@Scale (2016-19) – Advancing Care Coordination and Telehealth at Scale. Available from: https://www.act-at-scale.eu/

6. Monterde D, Vela E, Clèries M, grupo colaborativo GMA. Los grupos de morbilidad ajustados: nuevo agrupador de morbilidad poblacional de utilidad en el ámbito de la atención primaria. Atención Primaria. 2016;48:674–82.

7. Dueñas-Espín I, Vela E, Pauws S, Bescos C, Cano I, Cleries M, et al. Proposals for enhanced health risk assessment and stratification in an integrated care scenario. BMJ Open. 2016;6:e010301.
