# Supplementary material for: Evaluation of integrated care services in Catalonia: population-based and service-based real-life deployment protocols
Source: BMC Health Serv Res. 2019 Jun 11;19:370. doi: 10.1186/s12913-019-4174-2 (PMC6560864; doi:10.1186/s12913-019-4174-2)
Supplement: Supplementary file 4 — Table S4. Three interventions addressed to frail complex chronic patients. (DOCX 35 kb) [file 12913_2019_4174_MOESM4_ESM.docx]

**Additional file 4:** **TABLE S4.** **Three interventions addressed to frail complex chronic patients**

The table shows the detailed proposed evaluation for the community-based care for the frail elderly protocol according to the elements and dimensions described in the main text.

| **Objective** | | 1. To assess health value generation of three interventions for frail complex chronic patients (CCP) carried out in BSA 2. To generate a roadmap for regional scalability of the service |
| --- | --- | --- |
| **Study design** | | Prospective controlled cohort study (1:1 ratio) using propensity score matching |
| **Study subjects** | | Early discharge (n= 144): CCP acutely hospital admitted and promptly discharged to receive home-based post-acute care and/or rehabilitation;  Home-based Case Management (n= 566): CCP receiving home-based long-term care by a case management nurses;  Geriatric residences (n= 920): CCP receiving acute support, post-acute or continued care for elderly living in geriatric residences.  Controls (1:1 ratio for each service, n=1,630 in each study arm) will include patients receiving usual care within the geographical area of Badalona  A subgroup analysis of deeply characterized patients (n= 250 in each study arm) will be undertaken |
| **Inclusion criteria** | | CCP criteria (Age ≥ 65 yrs.; ≥ 2 chronic diseases; polypharmacy) for all three interventions  Home-based Case Management and Early Discharge: CCP and ≥ 3 hospital/ED admissions in the last year; having suffered a recent acute event  Geriatric residences: admitted in one of the residences of the BSA territory |
| **Exclusion criteria** | | Any neurological disease (e.g. severe-phase dementia with global deterioration scale ≥ 7) (18) or psychiatrically severe enough not to allow the subject to respond to questionnaires; subjects who do not agree to participate in the study. |
| **Variables & measurement tools** | **Health and well-being** | Mortality rate, avoidable hospital admissions, total bed days, 30-day readmissions, number of ER visits in the month  In the subgroup analysis (n= 250 in each arm)(*): Physical function (Katz-15 (1)); psychological well-being (MHI-5 (2)); social relationships & participation (IPA (3)); enjoyment of life (ICECAP-O (4)); resilience (BRS (5)); autonomy (Pearlin Mastery Scale (6)) |
|  | **Patient experience** | (*) Person centeredness (P3CEQ (7)); continuity of care (NCQ (8)); burden of medication (LMQ (9)); burden of informal caregiving (Informal care questionnaire (10)) |
|  | **Costs analysis** | Operational / running costs will be analysed for the following categories: staff, pharmacological and non-pharmacological treatment, consumables, diagnostic tests and procedures, transport, catering. Also, structural costs will be included as a separate category. |
|  | **Staff engagement** | Questionnaires for managers and health professionals from (11) |
| **Statistical analysis** | | Propensity score matching using age, sex, GMA (12,13), socioeconomic status, number of hospitalisations during the previous year and polypharmacy as matching variables. Health delivery assessment analyses comparing intervention and control groups. Identification of variables with predictive value. |
| **Expected outcomes** | | Assessment of health value generation of the service alongside a MCDA (14,15) analysis; to determine factors modulating success of the implementation strategy, including key performance indicators determination for service assessment |
| **Health risk assessment** | | Refine current health risk assessment criteria using population-based tools (GMA) |
| **Digital supporting tools** | | Only in selected cases: 1) Online Rehabilitation programme (Sword Health); and 2) Internet-based cognitive-behavioural therapy (Overcome your depression). |
| **Co-design activities** | | Several co-design activities involving all relevant stakeholders (policy makers, clinicians, third sector, end users and relatives), covering: 1) Identification of needs; 2) Service design; and 3) Evaluation of service. |
| **Future developments** | | Roadmap for scalability of the services at regional level |

BSA: Badalona Serveis Assistencials; CCP: complex chronic patients; GMA: Adjusted Morbidity Groups, population-based health risk assessment tool; ICECAP-O: ICEpop Capability Measure for Older People Questionnaire; IPA: Impact on Participation and Autonomy Questionnaire; LMQ: Living with medicines questionnaire; MCDA: Multi-criteria decision analysis; MHI-5: Mental Health Inventory Questionnaire; NCQ: Nijmegen Continuity of Care Questionnaire; P3CEQ; Person Centred Coordinated Experience Questionnaire; PAM-13: Patient Activation Measure questionnaire.

**References:**

1. Laan W, Zuithoff NPA, Drubbel I, Bleijenberg N, Numans ME, De Wit NJ, et al. Validity and reliability of the Katz-15 scale to measure unfavorable health outcomes in community-dwelling older people. J Nutr Health Aging. 2014;18:848–54.

2. Brazier JE, Harper R, Jones NM, O’Cathain A, Thomas KJ, Usherwood T, et al. Validating the SF-36 health survey questionnaire: new outcome measure for primary care. BMJ. 1992;305:160–4.

3. Cardol M, de Haan RJ, van den Bos GAM, de Jong BA, de Groot IJM. The development of a handicap assessment questionnaire: the Impact on Participation and Autonomy (IPA). Clin Rehabil. 1999;13:411–9.

4. Coast J, Peters TJ, Natarajan L, Sproston K, Flynn T. An assessment of the construct validity of the descriptive system for the ICECAP capability measure for older people. Qual Life Res. 2008;17:967–76.

5. Smith BW, Dalen J, Wiggins K, Tooley E, Christopher P, Bernard J. The brief resilience scale: Assessing the ability to bounce back. Int J Behav Med. 2008;15:194–200.

6. Pearlin LI, Schooler C. The structure of coping. J Health Soc Behav. 1978;19:2–21.

7. P3CEQ. Person centred coordinated experience questionnaire. Available from: http://p3c.org.uk/prom-detail/29

8. Uijen AA, Schellevis FG, van den Bosch WJHM, Mokkink HGA, van Weel C, Schers HJ. Nijmegen Continuity Questionnaire: Development and testing of a questionnaire that measures continuity of care. J Clin Epidemiol. 2011;64:1391–9.

9. Krska J, Morecroft CW, Rowe PH, Poole H. Measuring the impact of long-term medicines use from the patient perspective. Int J Clin Pharm. 2014;36:675–8.

10. Hoefman R, Van Exel N, Brouwer W. iMTA valuation of informal care questionnaire (iVICQ). version 1.0. Rotterdam: iBMG / iMTA. 2011.

11. ACT@Scale (2016-19) – Advancing Care Coordination and Telehealth at Scale. Available from: https://www.act-at-scale.eu/

12. Monterde D, Vela E, Clèries M, grupo colaborativo GMA. Los grupos de morbilidad ajustados: nuevo agrupador de morbilidad poblacional de utilidad en el ámbito de la atención primaria. Atención Primaria. 2016;48:674–82.

13. Dueñas-Espín I, Vela E, Pauws S, Bescos C, Cano I, Cleries M, et al. Proposals for enhanced health risk assessment and stratification in an integrated care scenario. BMJ Open. 2016;6:e010301.

14. Tsiachristas A, Cramm JM, Nieboer A, Rutten- van Mölken M. Broader Economic Evaluation Of Disease Management Programs Using Multi-Criteria Decision Analysis. Int J Technol Assess Health Care. 2013;29:301–8.

15. Rutten-Van Mölken M, Leijten F, Hoedemakers M, Tsiachristas A, Verbeek N, Karimi M, et al. Strengthening the evidence-base of integrated care for people with multi-morbidity in Europe using Multi-Criteria Decision Analysis (MCDA). BMC Health Serv Res. 2018;18:576.
