# Supplementary material for: Evaluation of integrated care services in Catalonia: population-based and service-based real-life deployment protocols
Source: BMC Health Serv Res. 2019 Jun 11;19:370. doi: 10.1186/s12913-019-4174-2 (PMC6560864; doi:10.1186/s12913-019-4174-2)
Supplement: Supplementary file 5 — Figure S1- Digital health framework in Catalonia (IS3). (DOCX 75 kb) [file 12913_2019_4174_MOESM5_ESM.docx]

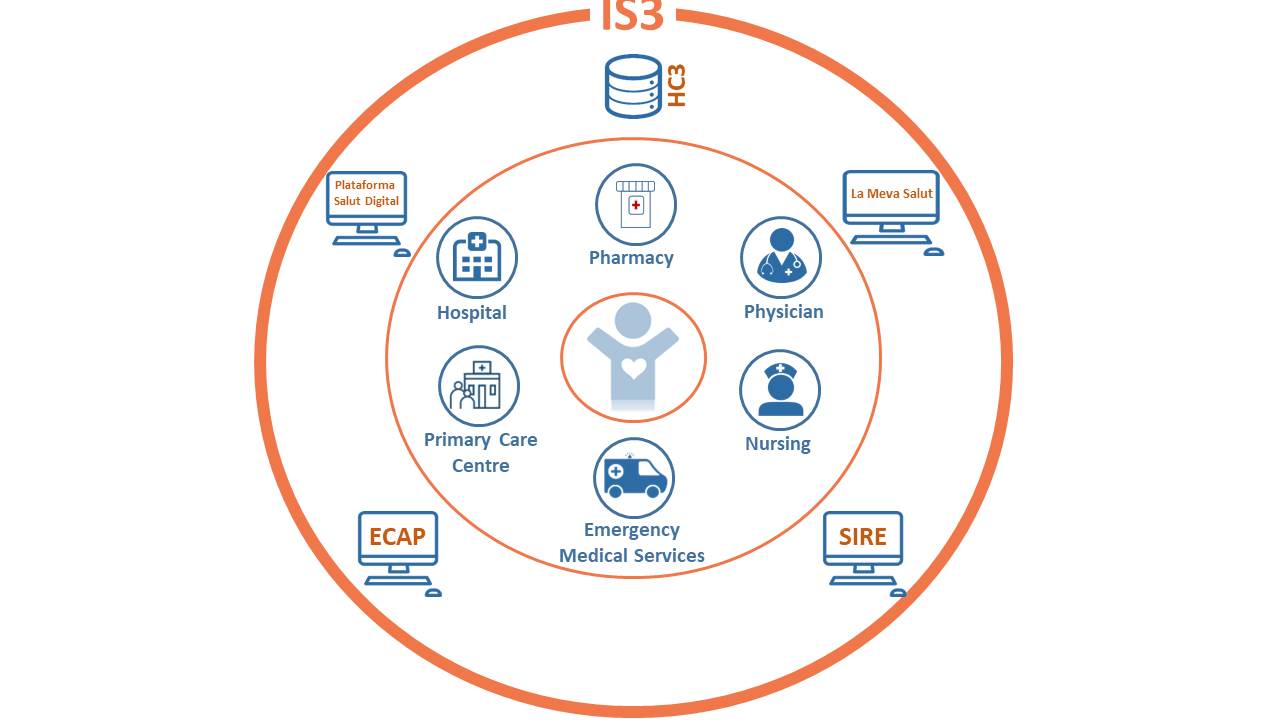


**Additional file 5:** ***Figure S1*** *- Digital health framework in Catalonia (IS3). Digital Health apps, wearables and/or medical devices are accredited to store and/or retrieve information from the Digital Health Platform (i.e., Plataforma Salut Digital), which acts as a repository of patient-generated health data, and in turn is interoperable with the regional personal health folder (La Meva Salut), the shared electronic health record of Catalonia (HC3), the regional health information system for primary care (ECAP), the regional electronic prescription system (SIRE), and/or other provider-specific health information systems.*
