# Supplementary material for: Evaluation of integrated care services in Catalonia: population-based and service-based real-life deployment protocols
Source: BMC Health Serv Res. 2019 Jun 11;19:370. doi: 10.1186/s12913-019-4174-2 (PMC6560864; doi:10.1186/s12913-019-4174-2)
Supplement: Supplementary file 6 — Annex 1 - Method for ASsessment of Telemedicine (mini-MAST). (DOCX 22 kb) [file 12913_2019_4174_MOESM6_ESM.docx]

# Additional file 6: Annex I - Method for ASsessment of Telemedicine (mini-MAST)

The annex depicts the different aspects to consider when evaluating a service in which telemedicine will be used.

| This checklist is completed by (name, title, department): |
| --- |
|  |

**Overview**

| 1. State the name or title of the telemedicine service. | | | | | | |
| --- | --- | --- | --- | --- | --- | --- |
|  | | | | | | |
| 2. Describe the purpose of implementing the telemedicine service. (Include descriptions of the patients/citizens, current technology and outcome measures). | | | | | | |
|  | | | | | | |
| 1. Write a short summary of the expected outcomes of the telemedicine service. | | | | | | |
|  | | | | | | |
| 1. Describe the evidence base for the assessment – e.g. a pilot study or a systematic literature review. | | | | | | |
|  | | | | | | |
| 1. Describe the evidence level of your current knowledge of the outcomes of the telemedicine service. | | | | | | |
| Evidence Level: | **1** | **2** | **3** | **4** | **5** |  |
| Clinical effectiveness |  |  |  |  |  |  |
| Patients’/citizens’ perspective |  |  |  |  |  |  |
| Economic aspects |  |  |  |  |  |  |
| Organisational aspects |  |  |  |  |  |  |

| 1. Describe the degree to which the outcomes described in the current evidence base can be transferred to the patients/citizens affected by the telemedicine service. |
| --- |
|  |

**TECHNOLOGY**

| 1. Which IT-technologies and equipment are included in the telemedicine service? |
| --- |
|  |
| 1. Describe the requirements on the technical infrastructure at the hospital, in the municipality, at the general practitioner’s office and in the patients’ or citizens’ homes. |
|  |
| 9. Describe the degree of planned integration into other IT-systems. |
|  |
| 10. Are there alternative vendors or other applicable telemedicine solutions available? |
|  |

**SAFETY**

| 11. Describe the experiences concerning the technical reliability e.g. number of patients’/citizens who can be reached by the IT-solution. |
| --- |
|  |
| 12. Describe the risks for patients/citizens when using the telemedicine service, e.g. health care errors/clinical incidents. |
|  |

**CLINICAL EFFECTIVENESS**

| 1. Describe the impact of the telemedicine service on the patients’/citizens’ health (e.g. mortality, morbidity, functionality, health related quality of life, pain). |
| --- |
|  |
| 1. Describe the impact of the telemedicine service on the patients´/citizens’ use of other health services. (e.g. hospitalisation, outpatient visits, visits to general practitioners, home nursing, assistance, rehabilitation). |
|  |

**PATIENT PERSPECTIVES**

| 1. How does the telemedicine service affect the patients´ perception of satisfaction and safety during treatment? |
| --- |
|  |
| 1. Estimate the percentage of patients complying with the inclusion criteria, who are expected to accept the telemedicine service. |
|  |
| 1. Describe the impact of the telemedicine service on the patients’/citizens’ self-care |
|  |

**ECONOMIC ASPECTS**

| 1. How many patients/citizens are expected to use the telemedicine service each year? |
| --- |
|  |
| 1. Describe increases and decreases in staff cost per year. |
|  |
| 1. Describe investment costs for equipment, adjustment of the physical environment, training, etc. |
|  |
| 1. Describe additional increases or decreases in cost per year (e.g. equipment, service agreements, support, integration, maintenance, transport). |
|  |
| 1. Describe expected changes in reimbursement per year for the institution offering the telemedicine service (e.g. DRG payment, municipal co-financing, payment for services). |
|  |

| 1. Calculate total cost and changes in reimbursement per year by inserting the information from questions 19-22 in the table below. | | | |
| --- | --- | --- | --- |
|  | 2018 | 2019 | 2020 |
| Staff (question 19) |  |  |  |
| Investment (question 20) |  |  |  |
| Equipment (question 21) |  |  |  |
| Service agreement (question 21) |  |  |  |
| Support (question 21) |  |  |  |
| Integration (question 21) |  |  |  |
| Maintenance (question 21) |  |  |  |
| Transportation (question 21) |  |  |  |
| **Total costs** |  |  |  |
| **Change in revenue ( question 22)** |  |  |  |
| **Overall result** |  |  |  |
| 1. Describe uncertainties in the above calculations. | | | |
|  | | | |
| 1. Describe the economic effects to be expected by other hospitals, municipalities, general practitioners, patients/citizens, relatives, etc. | | | |
|  | | | |

**ORGANIZATIONAL ASPECTS**

| 1. Describe the consequences for the staff of the telemedicine service, e.g. workflow, staff training and work environment. |
| --- |
|  |
| 1. Describe the need for chances in the physical environment when using the telemedicine service. |
|  |
| 1. Describe the consequences of the telemedicine service on other departments, support functions, hospitals, municipalities, general practitioners etc., concerning task shifts between professions and sectors. |
|  |

**SOCIO-CULTURAL, ETHICAL AND LEGAL ASPECTS**

| 1. Has the telemedicine equipment been CE marked – and if so in which class? |
| --- |
|  |
| 1. Has the legal basis for the telemedicine service been clarified, e.g. concerning patients’ rights or responsibilities? |
|  |
| 1. Which ethical or psychological considerations are raised by the telemedicine service, e.g. access to treatment, equality in treatment or waiting time for treatment? |
|  |
| 1. How is the patients’/citizens’ social or employment-related situation affected by the telemedicine service? |
|  |

**REFERENCES**

| 1. Insert your most important references documenting the effects of the telemedicine service. |
| --- |
|  |
